# Extracellular Vesicles: Versatile Nanomediators, Potential Biomarkers and Therapeutic Agents in Atherosclerosis and COVID-19-Related Thrombosis

**DOI:** 10.3390/ijms22115967

**Published:** 2021-05-31

**Authors:** Adriana Georgescu, Maya Simionescu

**Affiliations:** Institute of Cellular Biology and Pathology ‘Nicolae Simionescu’ of the Romanian Academy, 050568 Bucharest, Romania; adriana.georgescu@icbp.ro

**Keywords:** atherosclerosis, thrombosis, cardiovascular disease, COVID-19, extracellular vesicles, exosomes, microvesicles

## Abstract

Cells convey information among one another. One instrument employed to transmit data and constituents to specific (target) cells is extracellular vesicles (EVs). They originate from a variety of cells (endothelial, immune cells, platelets, mesenchymal stromal cells, etc.), and consequently, their surface characteristics and cargo vary according to the paternal cell. The cargo could be DNA, mRNA, microRNA, receptors, metabolites, cytoplasmic proteins, or pathological molecules, as a function of which EVs exert different effects upon endocytosis in recipient cells. Recently, EVs have become important participants in a variety of pathologies, including atherogenesis and coronavirus disease 2019 (COVID-19)-associated thrombosis. Herein, we summarize recent advances and some of our own results on the role of EVs in atherosclerotic cardiovascular diseases, and discuss their potential to function as signaling mediators, biomarkers and therapeutic agents. Since COVID-19 patients have a high rate of thrombotic events, a special section of the review is dedicated to the mechanism of thrombosis and the possible therapeutic potential of EVs in COVID-19-related thrombosis. Yet, EV mechanisms and their role in the transfer of information between cells in normal and pathological conditions remain to be explored.

## 1. Introduction

Over the course of evolution, the communities of cells of all organisms have found means to converse and communicate their physiological or pathological state with each other, reminding us of the well-known promise, “for better for worse, … in sickness and in health, …till death us do part” (marriage vows in the Catholic church).

Among the various methods of communication, the most recently discovered common instruments are extracellular vesicles (EVs), an assembly of cell-derived vesicles of different origins and sizes encompassing endosome-derived exosomes, plasma membrane-derived microvesicles (ectosomes) and apoptotic bodies.

EVs are released from activated or apoptotic cells carrying biological molecules such as DNA, mRNA, microRNA (miRNA) and others as cargo. They play a particularly important role in cell–cell communication and signaling due to their ability to transport and transfer their cargo to recipient cells. Several research groups, including ours, have uncovered the capacity of EVs to function as communicators, signaling mediators, biomarkers and therapeutic agents in atherosclerosis and thrombosis [1,2,3,4,5,6]. In addition, EVs have been implicated in varied pathologies such as neurodegenerative diseases [7] and metastasis [8,9]. This review will focus on the role of EVs in atherosclerosis and thrombosis, the major causes of death in developed countries and major public health problems.

Atherosclerosis affects the arterial tree, in particular, the aorta and the cerebral, carotid, coronary, iliac and femoral arteries at the level of which the plaques (atheroma) develop in the intima of the vessel wall. In humans, the first lesions may appear early in life, while atherogenesis develops over the years. However, the slow or fast progression of the lesions depends on traditional risk factors such as dyslipidemia, hypertension, stress, genetic factors and others.

The process of atherogenesis involves a complex scenario in which cellular and molecular inflammatory and immunomodulatory pathways intersect each other to develop, in several stages, the final atherosclerotic plaque [10,11,12]. Recent studies have been focused on the identification of new compounds that could be used either as biomarkers or targets for new therapies to stop the evolution of atherosclerotic plaques. In this regard, the discovery of the presence and determining the role of EVs in all stages of plaque formation was key.

We present here the pathophysiology of atherosclerosis, focusing on the cells and molecules involved, immune cell activation and inflammatory processes, the implication of EVs in the atheroma progression, and highlight promising new directions involving EVs as biomarkers and therapeutic agents.

Thrombosis is a common occurrence in atherosclerosis; it is a complex process in which blood-activated platelets and numerous coagulation factors contribute to thrombus formation in the arterial wall (arterial thrombosis) or veins (venous thrombosis). Once formed, the thrombus blocks or completely obstructs blood flow, causing major complications, especially in the brain, heart or lungs. A high rate of thrombotic events occurs in coronavirus disease-2019 (COVID-19) patients, a current major global problem caused by severe acute respiratory syndrome coronavirus-2 (SARS-CoV-2).

Here, we will discuss novel data regarding the role of EVs in COVID-19-generated thrombotic events and the potential of EV-based therapies as a new/additional remedy for this disease.

## 2. Extracellular Vesicles: Our Current Understanding

### 2.1. Terminology and Biogenesis Pathways of the Distinct Extracellular Vesicle Population

*Extracellular vesicles (EVs)* is a term used to describe a heterogeneous group of vesicles located in different types of tissues or biological fluids such as blood, urine, saliva, breast milk and the amniotic, cerebrospinal, synovial, seminal fluid and bronchial lavage [13,14,15,16,17].

All cells, prokaryotes and eukaryotes produce and release EVs as a normal physiological process, and also in pathological conditions as a result of their activation or apoptosis. Based on their size, morphology and biogenesis, EVs have been classified as either exosomes and ectosomes, with the latter being called also microvesicles (MVs) or microparticles (MPs). In addition, apoptotic bodies are considered to be part of the EV family [18], although they differ in content and function.

*Exosomes* were defined in 1981 as “exfoliated membrane vesicles” [19]. Subsequently, an ultrastructural study revealed that vesicles measuring approximately 50 nm were exocytosed from multivesicular bodies (MVBs) [20]. Recent studies defined exosomes as EVs with a size range of about 40 to 160 nm (average ~100 nm) in diameter, and as being of endosomal origin [21].

**Figure 1:**
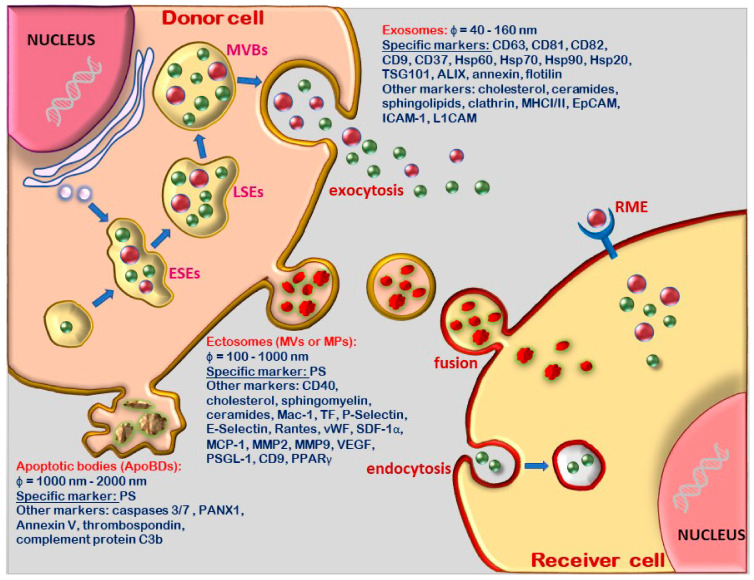
Diagram representing the classification, biogenesis, molecular content and interactions with target cells of extracellular vesicle (EVs). Exosomes originate from the early sorting endosome (ESE), which turn sequentially into late sorting endosome (LSE), and multivesicular bodies (MVBs), whose intraluminal vesicles (ILVs) are exocytosed upon fusion and inward invagination of the plasmalemma. Ectosomes, also named microparticles (MPs) or microvesicles (MVs) are formed upon plasma membrane budding with phosphatidyl serine (PS) on the outer membrane surface. Apoptotic bodies (ApoBDs are membrane-derived large vesicles originating from apoptotic cells with PS as surface marker). (RME: receptor mediated endocytosis).

As shown in Figure 1, exosomes are formed in a complex process originating from the early sorting endosome, which successively turn into late sorting endosome and MVBs. The latter contain intraluminal vesicles (ILVs) that, upon fusion with the plasma membrane, secrete ILVs as exosomes [21].

In the biogenesis of exosomes, the proteins involved include ESCRT (endosomal sorting complexes required for transport) proteins, Ras-related protein GTPase Rab (Rab7a and Rab27b), TSG101 (tumor susceptibility gene 101), Sytenin1, Syndecan1, ALIX (apoptosis-linked gene 2-interacting protein X), phospholipids, tetraspanins, ceramides, sphingomyelinases, SNARE proteins (SNAP Receptor) and others [22,23,24,25]. Further research is needed to understand the functions of these proteins in exosome biogenesis. The physiological role of exosome release is not completely understood, but it is assumed that they contribute to cellular homeostasis [26].

*Ectosomes or microvesicles (MVs)* were first described as small particles released from platelets [27]. Now, it is accepted that MVs are released in an evolutionarily-conserved manner in both prokaryotes and eukaryotes [28,29]. MVs have been defined as EVs with a size range of about 100 to 1000 nm in diameter. They are plasma-membrane-derived particles released into the extracellular space by the direct outward budding and fission of the plasmalemma taking within some of the cytosolic content and membrane receptors of the paternal cell [30] (Figure 1). MV release implies the contribution of Ca^2+^-dependent enzymatic interactions such as aminophospholipid translocases (floppase or scramblase), which cause the asymmetrical rearrangement of plasma membrane phospholipids and subsequent membrane curvature that favors vesicle budding [31,32]. More specifically, the increase in the intracellular Ca^2+^ levels leads to the redistribution of plasma membrane phospholipids, causing phosphatidylserine (PS) exposure on the outer face of the membrane, disarrangement of the cytoskeletal proteins and finally, MV release [33]. The detachment of MVs from donor cells involves the contraction of cortical actin beneath the plasma membrane due to high levels of intracellular Ca^2+^ [34]. Also, it has been shown that MV release is regulated not only by membrane lipid microdomains, but also by regulatory proteins such as ADP-ribosylation factor 6 (ARF6) [35,36].

Although research on EVs is constantly evolving, the classification of EVs is still as either exosomes and ectosomes or MVs. However, it is important to note that many publications include apoptotic bodies (ApoBDs) or apoptosomes in the EV group.

*ApoBDs* are larger vesicles released from dying cells with sizes ranging from 1000 nm to 2000 nm that, under specific conditions, can be more abundant than exosomes or MVs [18].

ApoBDs are released during the early stages of apoptosis upon rearrangement of membrane lipids that induce PS translocation from the inner to the outer leaflet and a subsequent release of ApoBDs into the extracellular space [37] (Figure 1). The external translocated PS binds to Annexin V, which is recognized by phagocytes, and ApoBDs undergo phagocytosis [38]. These apoptosis-derived large cellular fragments, that are taken-up by neighboring cells (macrophages, parenchymal cells or neoplastic cells), are degraded within phagolysosomes or recycled; therefore, they cannot be regarded as EVs which are involved in intercellular communication.

### 2.2. Molecular Content and Biological Functions of Extracellular Vesicles

Depending on, or because of, the different cellular origin of EVs, differences in the protein and lipid composition exist between exosomes and MVs, on the basis of which they exert specific biological functions.

*Exosomes* have several cell membrane proteins on their surface, such as tetraspanins, integrins and immunomodulatory proteins, and as cargo, have cell cytoplasmic proteins, i.e., RNA, DNA, amino acids and metabolites. As depicted in Figure 1, the most common proteins used as biomarkers for exosomes are tetraspanins (CD63, CD81, CD82, CD9 and CD37), heat-shock proteins (Hsp60, Hsp70, Hsp90 and Hsp20), tumor susceptibility gene (TSG101), annexin, flotillin and apoptosis-linked gene 2-interacting protein X (ALIX) [18]. Exosomes may contain and transport DNA, mRNAs, miRNAs, pre-miRNAs and other noncoding RNAs that are transferred to recipient cells and tissues [39,40], and thus, function in intercellular communication and signaling [41,42,43].

In addition, specific transmembrane proteins are present on the exosome surface, i.e., epithelial cell adhesion molecule (EpCAM), epidermal growth factor receptors (EGFRs), lymphocyte function-associated antigen 1 (LFA-1) integrin, intercellular adhesion molecule-1 (ICAM-1, known as CD54), L1 cell adhesion molecule (L1CAM) and endoglin (CD105). The latter is a transforming growth factor β (TGF-β) receptor and integrin ligand which plays a key role in vascular pathology, angiogenesis, inflammation and hemostasis [44]. It has been shown that in hereditary hemorrhagic telangiectasia (HHT), a disease caused by mutations in the endoglin gene, patients infected with SARS-CoV-2 suffer milder symptoms with lower clinical impact than the general population [45].

The presence of the above molecules point to the cellular origin of exosomes, and may explain their capacity to adhere and fuse with the plasma membrane of recipient cells [46].

As a result of this molecular composition, several pathways have been proposed for exosomes interaction with target cells: (1) direct fusion with the cell plasmalemma; (2) adhesion to the cell surface by receptor-ligand interaction; (3) paracrine signaling as a result of the release of the exosome content generated by the destabilization of their membrane under low pH conditions, and (4) endocytic uptake [47,48] (Figure 1).

*Microvesicles (MVs)* express numerous features of the donor cell including specific surface antigens and receptors [49]. Thus, endothelial cell-derived microvesicles (EMVs) express the speciflc protein CD144 on their external leaflet, platelet-derived microvesicles (PMVs) released from activated platelets express specific protein CD41 [2], and leukocyte-derived microvesicles (LMVs) express as specific marker CD18 (integrin beta 2) [5]. All types of MVs have PS on the outer face of plasmalemma, as a common specific marker. Thus, the specific molecular signature of MVs consists of several surface proteins which are specific to paternal cells and PS as a key identifier [28,33] (Figure 1). PS-positive MVs can be easily distinguished from exosomes through their capacity to bind annexin V or lactadherin [50,51,52].

MVs are also rich in the surface marker CD40, as well as cholesterol, sphingomyelin and ceramides [47] (Figure 1). Furthermore, MVs carry the original cell-specific membrane proteins on their surface, such as macrophage integrin-1 (Mac-1), tissue factor (TF), P-Selectin, E-Selectin, Rantes or CCL5 (chemokine (C-C motif) ligand 5), stromal cell-derived factor 1 α (SDF-1α), P-Selectin glycoprotein ligand 1 (PSGL-1), CD9 and endoglin [53] In addition, they carry proteins derived from the cytoplasm of the cells of origin: von Willebrand factor (vWF), monocyte chemoattractant protein-1 (MCP-1), matrix metallopeptidases (MMP2, MMP9), vascular endothelial growth factor (VEGF), DNA, mRNAs, miRNAs, noncoding RNAs and peroxisome proliferator-activated receptor gamma [46,54,55,56,57,58] (Figure 1).

MVs released into biological fluids or tissues have the ability to interact with target cells and transfer their abundant and complex biological content, potentially affecting their function. The most significant changes induced by MVs on recipient cells are those caused by the release of the miRNAs, mRNAs and DNA they contain [46]. The genetic material transferred into target cells regulates the gene expression and protein synthesis of the recipient cells, influencing their biological characteristics. MV-target cell interaction occurs through several pathways: (1) specific receptor–ligand interactions affect different intracellular signaling pathways; (2) direct fusion to the cell plasma membrane, and (3) endocytic uptake [59,60] (Figure 1).

*Apoptotic bodies or apoptosomes* are variable in size, structure, composition, and their specific identification marker is PS. They differ from MVs by the presence of caspases 3 and 7 and their substrates (e.g., Pannexin1 (PANX1), ROCK1), and of Annexin V, thrombospondin and complement protein C3b [38,61] (Figure 1).

In summary, due to their variable size, diverse cellular origin and varied biological content, EVs (exosomes and ectosomes) can be regarded as a highly heterogeneous population with multiple functionalities which are able to mediate complex cell-to-cell communication over short or long distances.

Importantly, in various diseases, the detection of EVs in body fluids (liquid biopsies) offers a window into altered cellular or tissue states, and provides a multicomponent diagnostic readout. The trafficking and efficient exchange of cellular components through EVs has led to their use in the design of EV-based therapeutics.

## 3. Pathophysiology of Atherosclerosis

Atherosclerosis is an inflammatory disease characterized by the narrowing of the lumen of blood vessels due to the deposition of cholesterol-rich plaques in the arterial wall. Atheroma formation entails a progressive process in which the gradual implication of various cells and their secretory products define a sequence of events that leads from the fatty streak to fibro-lipid plaque, and ultimately, to plaque rupture and atherothrombosis.

### 3.1. Consecutive Stages, the Cells and Molecules Involved in the Formation of Atheroma

**Figure 2:**
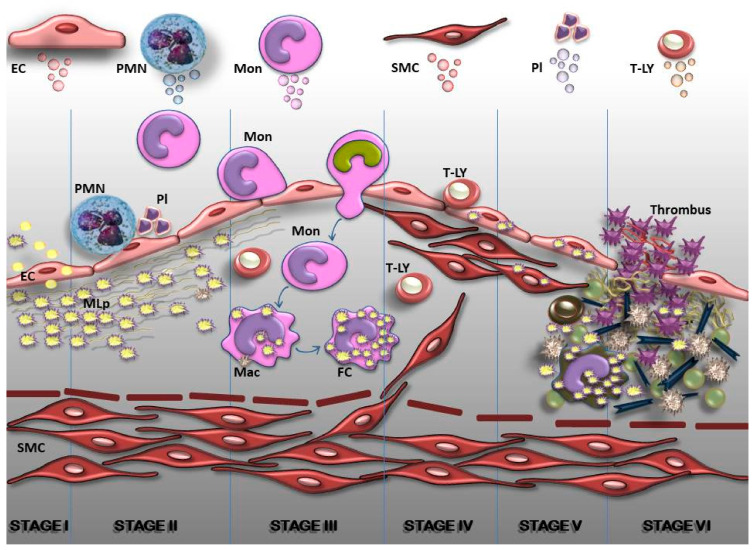
Diagram representing the arbitrary consecutive stages in the development of atherosclerotic plaques in arterial lesion-prone areas. *Stage I. Endothelial cell (EC) activation/modulation of constitutive functions.* The initial stage in atheroma formation is the accumulation within the plasma of oxidatively modified lipoproteins (MLp) that induce EC activation and increased transcytosis of MLp, i.e., their housing in the subendothelium where they interact with the components of the extracellular matrix (ECM). EC switch to a secretory phenotype that produces an excess of hyperplasic basal lamina. *Stage II. EC dysfunction.* Affected luminally by the alterations of plasma homoeostasis and abluminally by the accrual of MLp, the EC initiate an inflammatory process (i.e., the expression of new or more cell adhesion molecules, cytokines and chemokines) that attract immune cells such as neutrophils (PMN) which interact with platelets (Pl) and assist leucocyte migration. *Stage III. Commencement of a robust inflammatory reaction.* The recruitment of blood monocytes (Mon) and T lymphocytes (T-LY), diapedesis of Mon into the intima, where they become activated macrophages (Mac), expressing scavenger receptors which function in the uptake of MLp and the formation of foam cells (FC). Lymphocytes switch to activated pro-inflammatory (Th 1) and anti-inflammatory (Th 2 and TREG) cells that secrete cytokines and chemokines. *Stage IV. SMC-key participants in the formation of fibrous plaques.* The proliferation of resident SMC from the intima, and of SMC which has migrated from the media to the intima leads to the formation of a fibrous cap that is accompanied by increased synthesis of ECM. *Stage V. Resident and immune cells and the factors they secrete generate a calcified fibro-lipid plaque.* SMC, macrophages-derived foam cells, apoptotic cell-derived lipids and calcification centers form a necrotic core rich in cholesterol crystals. *Stage VI. The unstable fibro-lipid plaque.* Thinning of the fibrous cap, EC apoptosis and the accumulation of pro-inflammatory mediators leads to the physical rupture of the plaque. The direct contact between the ECM, the tissue factors and circulating platelets and blood coagulation components trigger thrombosis. In the upper part of the figure, the main cell sources of extracellular vesicles are shown.

Although continuous, the atherogenic process can be arbitrarily divided into six main stages: (1) endothelial cell (EC) activation on account of the accumulation of plasma oxidatively-modified, low-density lipoproteins (MLp) that induce changes in EC constitutive function (i.e., transcytosis and subendothelial MLp accumulation); (2) EC dysfunction manifested by the expression of novel cell adhesion molecules; (3) robust inflammation, i.e., recruitment of the circulating immune cells; (4) migration of smooth muscle cells (SMCs) from the media to intima, forming the fibrous plaque; (5) the development of the calcified fibro-lipid plaque made of apoptotic resident and immune cells, and calcium deposition that leads to (6) the unstable fibro-lipid plaque, and the ensuing rupture, exposure of the vascular extracellular matrix, platelet adherence and the formation of thrombus [12,62,63,64,65] (Figure 2).

The earliest event occurring at the inception of atherosclerosis is the increase in plasma concentration, transport and deposition within the subendothelial space of the biochemically-modified and oxidized low-density lipoproteins [62]. This change in the microenvironment induces EC activation which modifies constitutive function, e.g., increasing permeability. Upon transcytosis across the endothelium, the MLp accumulated within the subendothelium interact with the extracellular matrix, and possibly as a security mechanism, a hypertrophic basal lamina develops. At this point, the EC are exposed on both the apical and basal surface to the aggressive oxidized MLp. This leads to EC dysfunction, manifested by a defense reaction, i.e., the surface expression of adhesion molecules, including ICAM-1 (Intercellular Adhesion Molecule-1), VCAM-1 (Vascular-cell Adhesion Molecule-1), E and P-Selectins and others. These changes are the start of a robust inflammatory reaction. Circulating neutrophils adhere to the EC surface, interact with plasma platelets and trigger the recruitment of monocytes and lymphocytes. Both SMCs and EC secrete chemoattractant cytokines like MCP-1 (monocyte chemotactic protein-1) and M-CSF (monocyte colony stimulating factor), providing a milieu for the adhesion of monocytes and T-lymphocytes to the endothelium and their subsequent diapedesis into the intima of the vessel wall.

In the intima, migrated monocytes differentiate into activated macrophages that express scavenger receptors, take up oxidized MLp, become overloaded with lipids and turn into macrophage derived-foam cells.

Besides monocytes-derived macrophages, immune cells, including dendritic cells, mast cells, T cells and B cells, also infiltrate the intima and adventitia of the atherosclerotic plaque [66,67].

The foam cells secrete cytokines such as platelet-derived growth factor (PDGF), interleukin-1 (IL-1), interferon gamma (IFNγ), tumor necrosis factor α (TNFα) and TGF-β that stimulate SMC proliferation and migration to form the fibrous cap. The SMCs synthesize abundant extracellular matrix proteins, in particular, collagen and fibronectin, which contribute to strengthening the fibrous cap.

With time, the atherosclerotic plaque, formed by macrophage-derived foam cells, lipids, calcification centers and cell debris, becomes vulnerable. The macrophages and T lymphocytes that are localized preferentially at the edges of the plaque secrete matrix metalloproteinases (MMP) that degrade extracellular matrix proteins and inhibit SMC proliferation and collagen synthesis, which weakens the fibrous cap, leading to its rupture [68].

A potential role of P-Selectin, E-Selectin, vWF, MMP2, MMP9, MMP12, TIMP1, TIMP2 and collagen type I and III in experimental atherosclerotic lesion development has been reported [69].

Atherosclerotic plaque rupture exposes the basal lamina constituents which attract circulating platelets, thereby initiating a coagulation process, leading to the formation of an obstructive thrombus at the rupture site. Under the shearing forces of the blood flow, the thrombus may detach, causing thromboembolism and myocardial infarction or stroke [70].

Not all atherosclerotic plaques are vulnerable and unstable, and evolve toward rupture. For reasons that remain unclear, some plaques are stable, while others pass from stable to unstable. In a stable plaque, a significant number of adaptive immune cells, including T and B lymphocytes, participate. Regulatory T (T-reg) cells that produce TGF-β and IL-10 inhibit antigen-specific activation of T helper 1 (Th-1) cells that produce IFNγ and regulate the inflammatory status of the monocytes-derived foam cells. The latter increase the synthesis of the anti-inflammatory cytokines (i.e., TGF-β, IL-10) and the collagen production in SMCs, thus preserving the thick fibrous cap. The cap of the stable plaque provides an effective barrier that prevents plaque rupture and exposure of lesion matrix (and prothrombotic factors), thereby diminishing the likelihood of thrombus formation and the ensuing clinical events [71].

Not all atherosclerotic plaques are a result of hypercholesterolemia. Often, an inflammatory reaction induced by putative antigens that stimulate T lymphocytes generates atherosclerotic plaques in the absence of systemic hypercholesterolemia [63]. One-half of all heart attacks and strokes occur in individuals without hypercholesterolemia, and one-fifth of all cardiovascular events take place in the absence of any of risk factors [63]. Thus, the process is more complex than previously thought. The traditional view on the role of dyslipidemia in the generation of atherosclerosis was founded upon evidence that inflammation alone could be a cause and/or intervenes in all stages of this disease.

### 3.2. Immune Cell Activation and Inflammatory Process in Atherogenesis

Inflammation is a defensive response against a diverse range of exogenous or endogenous factors, characterized initially by leukocyte accumulation in the sites of infection or damaged cells and tissues.

In general, inflammation is classified as acute, which is of short duration and is characterized by neutrophil accumulation, and chronic, which is of long duration and engages other leukocyte types such as monocytes/macrophages, T cells, eosinophils, basophils, mast cells and dendritic cells [72].

Chronic inflammation is specific to the process of atherogenesis, and all stages of the atherogenic process are accompanied by an inflammatory response. Activated, immunocompetent cells, including T lymphocytes and monocyte/macrophages, coexist in atherosclerotic lesions [73,74,75].

An essential link was reported between inflammation and thrombosis that is characterized by the occurrence of macrophages that produce TF, a major procoagulant that triggers thrombosis within atherosclerotic plaques [76]. Preclinical animal studies suggested that concomitant targeting of inflammatory and thrombotic pathways could produce substantial beneficial effects in cardiovascular diseases [77].

Recently, the inflammatory condition underlying the atherogenic process caused by metabolic impairment known as meta-inflammation has been closely linked to a dysregulation of trained immunity, i.e., the memory of innate immune cells. Although this training is an effective way to protect the body against recurrent infections, in the case of atherosclerosis, it accelerates and aggravates plaque development by the activation of the (Nod-like receptor protein 3) NLRP3 inflammasome [78,79]. This immune memory implies the rewiring of intracellular metabolic pathways and epigenetic reprogramming of histone modifications [80].

In monocytes and their bone marrow progenitors, metabolic and epigenetic reprogramming can induce trained immunity, which might contribute to the persistent nonresolving inflammation that characterizes atherosclerosis. In patients with established or increased risk of atherosclerosis, the occurrence of monocytes with trained immunity has been reported [80]. Thus, immune activation and inflammatory processes are decisive mechanisms in the pathogenesis of atherosclerosis, and as such, they are key therapeutic targets in cardiovascular disease.

Interestingly, genetic sequencing of whole blood-derived DNA revealed that clonal mutations in myeloid stem cells are associated with higher risks of cardiovascular events and hematopoietic malignancies. The clinical repercussions of this biological state, termed CHIP (clonal hematopoiesis of indeterminate potential), have been associated with increased risk of hematological malignancies and cardiac diseases such as atherosclerosis, myocardial infarction, aortic valve stenosis and congestive heart failure. Further studies are needed to better understand the complex relationship between CHIP and cardiovascular diseases [81].

## 4. Extracellular Vesicles Have the Potential to Predict, Monitor and Act as Therapeutic Agents in Atherosclerosis

### 4.1. Extracellular Vesicles of Various Cell Origin Are Implicated in All Stages of Atheroma Formation

EVs have been reported to be present in both developing plaques and in advanced plaques, suggesting that they participate throughout the atherogenic process, from the initial to the final stage. The EVs present in the fibro-atheromatous plaque originate from leukocytes (52%), followed by macrophages (29%), erythrocytes (27%), lymphocytes (15%), SMCs (13%) and endothelial cells (8%) [82]. EVs could play an important role in the transition from endothelial activation–dysfunction to the formation of atheroma.

*EVs and endothelial dysfunction.* In physiological conditions, EVs maintain the communication between the cells of the vessel wall. In pathological conditions, as in the presence of oxidized MLp, EVs have a negative effect on the vascular endothelium. For example, platelet-derived EVs activate pro-inflammatory cytokines IL-8, IL-1 and IL-6, that in turn stimulate the expression of VCAM-1, ICAM-1 and E-selectin, thus enhancing the adhesion properties of the endothelium and promoting the early development of atherosclerosis [70,83]. Moreover, platelet-derived MVs transfer the adhesion molecule CD41 to endothelial cells, conferring pro-adhesive properties upon them [84] and stimulating the proliferation and migration of the vascular SMCs from the media to the intima, thus increasing lesion progression [85]. We have shown that the systemic delivery of autologous platelet-derived MVs of pathological origin activates the platelets and the release of inflammatory mediators (SDF-1α, VEGF, RANTES, TF, TFPI, MCP-1, IL-6, IL-1ß, IL-8), and generates the accumulation of lipids, macrophages and more MVs into the liver and aorta [69,86]. In vitro assays have demonstrated that human SMC-derived exosomes may promote thrombogenesis [87].

Interestingly, all immune cell-derived EVs participate in endothelial cell dysfunction in atherosclerosis. Thus, T cell-derived EVs inhibit endothelial nitric oxide synthase (eNOS), generating an imbalance between circulating vasodilator and vasoconstrictor compounds, which, in turn, increase the oxidative stress and induce arterial stiffness. Monocyte-derived EVs promote the apoptosis of endothelial cells and SMCs, leading to major changes in endothelial permeability [85]. Macrophage-derived exosomes impair the growth of endothelial cells. The mechanism by which EVs induce impaired endothelial functions involves the inhibition of AKT/eNOS-heat shock protein 90 (Hsp90) and the p38 MAPK-dependent pathway [88].

*EVs and plaque stability/instability.* Reportedly, the EVs present in atherosclerotic plaques are involved in cellular signaling, atherosclerosis development, immune responses, inflammation, cell proliferation and migration, cell death and vascular remodeling during the progression of the disease [89]. One mechanism by which EVs contribute to the stability of the atherosclerotic plaque is their implication in vascular calcification. Specifically, once the atherosclerotic plaque is formed, SMCs and macrophages release the “calcifying” EVs that, being rich in calcium and phosphorus, promote mineralization [90].

Moreover, we have shown that platelet-derived MVs may contribute to the progression of atherosclerotic vascular disease by increasing the expression of surface receptors (TF, P-Selectin, SDF-1α, PSGL-1) and the protein content (vWF MCP-1, MMP2, MMP9, VEGF) of the vesicles [5].

The M1-like macrophage-derived EVs transfer integrins to SMCs that activate extracellular regulated protein kinase (ERK) and protein kinase B (Akt), which, in turn, accelerate ECM production and cell migration and adhesion, aggravating atherosclerosis [91]. In addition, macrophage-derived EVs induce SMC proliferation and migration by delivering miR-21-3p to activate phosphatase and tensin homolog (PTEN) in these cells [92]. These data show that EVs play key roles in SMC proliferation and migration within the plaque.

Angiogenesis is one of the mechanisms involved in plaque instability. Reportedly, macrophage-derived EVs promote angiogenesis by miR-150 transfer, consequently increasing plaque vulnerability to rupture [93]. Furthermore, endothelial cell-derived MVs promote angiogenesis through the transfer of proangiogenic molecules such as ligand CD40 (CD40L), growth factors and their activators [56,94].

Also, it has been reported that endoglin^+^ endothelium-derived MVs from chronically thromboembolic pulmonary hypertensive patients are likely to represent a protective mechanism facilitating endothelial cell survival and angiogenesis, and reduce the effects of vascular occlusion and endothelial damage [53].

### 4.2. Extracellular Vesicles as Nanomediators in Atherosclerosis

Recent findings have demonstrated that EVs act as biological vehicles which are directly involved in the transport of lipids such as cholesterol, fatty acids and eicosanoids. By transferring lipids between cells, EVs play a key role in triggering the atherogenic process. For example, adipose tissue-derived EVs increase oxLDL uptake in macrophages, thereby promoting the formation of foam cells [95]. One mechanism by which EVs from various cells induce cholesterol accumulation and formation of macrophage-derived foam cells is the activation of the toll-like receptor pathway [96]. In addition, the intracellular accumulation of cholesterol could be due to the presence of exosomes carrying miRNAs (i.e., miR-30e and miR-92a) that inhibit ABCA1 (ATP binding cassette A1) and ABCG-1 (ATP-binding cassette sub-family G member 1), the main transporters of cholesterol in the human body [97].

T cell-derived exosomes, containing a high cholesterol concentration, induce the production of TNFα and accelerate lipid deposition in the plaque [98]. Furthermore, endothelial cell-derived exosomes deliver the Notch ligand Delta-like 4 (DLL4) into target endothelial cells, inhibiting the Notch signaling pathway and altering angiogenesis [99].

Remarkably, under pressure overload or stretch conditions, cardiomyocyte–derived exosomes contain functional angiotensin II type 1 receptors, that, upon transfer to skeletal muscle cells, to other cardiomyocytes and mesenteric resistance vessels, augment the cellular response to angiotensin II, with severe functional consequences for the cardiovascular system [100].

Platelet-derived MVs also contribute to the acceleration of atherogenesis. They stimulate leukocyte–leukocyte interaction by binding their surface P-Selectin receptor to leukocyte PSGL-1, promoting the accumulation and infiltration of leukocytes into the intima [101]. In addition, platelet-derived MVs transfer the cytokine RANTES to endothelial cells and monocytes, inducing the release of pro-inflammatory cytokines such as IL-8, IL-1β, IL6 and TNFα, and the ensuing increased adhesion and infiltration of leukocytes [102,103]. Interestingly, the level of platelet-derived MVs has been positively correlated with the abnormal carotid intima-media thickness in obesity and other risk factors, suggesting their involvement in the changes occurring in the vascular wall in atherosclerosis. For instance, MVs containing arachidonic acid released after platelet activation have a procoagulant effect on endothelial cells [104].

Neutrophil-derived MVs (CD66b^+^) carrying miR-155 increase NF-κB expression in EC, promoting endothelial inflammation, monocyte recruitment and atherosclerotic plaque development in *ApoE^−/−^* mice subjected to a high-fat diet [105].

Last but not least, EV signaling plays an important role in the process of apoptosis. For example, T-cell-derived EVs promote macrophage apoptosis, and during the apoptotic signaling cascade, cause the release of new apoptotic messages that extend the process to neighboring cells [106]. Likewise, monocyte and endothelial cell-derived EVs encapsulating caspase-1 and 3 induce SMC apoptosis.

In general, the involvement of EVs in the pathophysiology of atherosclerosis is more complex than initially thought. Some studies have shown that EVs may have opposite roles, being involved either in the formation of atherosclerotic plaques or playing a protective role.

It is safe to assume that the contribution of EVs to the atherosclerotic process depends largely on their origin, the microenvironment, the stage of the process, the concentration of the vesicles and the presence or absence of risk factors such as hyperlipemia, hyperglycemia, hypertension or obesity.

### 4.3. Extracellular Vesicles as Biomarkers in Atherosclerosis

Since the content and function of the circulating EVs reflect the status of their cellular source, and their number increases significantly in pathological conditions, it is safe to assume that they could be reliable biomarkers to assess the stage of the disease or monitor the effect of drugs in various maladies.

Several studies have stated a direct correlation between the levels of circulating EVs and the cardiovascular events in patients with stable coronary artery disease. It has been shown that elevated levels of circulating endothelial cell-derived EVs are associated with endothelial dysfunction and can predict future coronary events [107,108]. In patients with stable coronary artery disease and cardiovascular risk factors (e.g., diabetes), increased levels of circulating endothelial cell-derived EVs were considered to be an independent risk factor for the outcome of the disease [109]. The levels of circulating EVs were found to be augmented in patients with acute coronary syndrome compared with those with stable angina [110]. Also, leukocyte-derived EVs are considered to be potential biomarkers to predict subclinical atherosclerosis and plaque vulnerability in patients with unstable carotid plaques [111,112].

Besides the increased number of EVs found in body fluids, changes of the cargo molecules in pathological conditions are considered potential biomarkers as well. In this sense, Goetzl et al., reported altered cargo proteins of human endothelial cell-derived exosomes in atherosclerotic cerebrovascular disease [113]. In these patients, the vesicles had normal size and marker proteins, compared to age- and gender-matched control subjects. However, these exosomes are enriched in VCAM-1, vWF, eNOS, platelet-derived growth factor (PDGF)-BB, angiopoietin-1 and lysyl oxidase-2, and the cerebrovascular-selective proteins glucose transporter 1, permeability-glycoprotein and large neutral amino acid transporter 1. The data underline the great potential of EVs and the cargo they carry for use as biomarkers in atherosclerosis [113,114].

The potential of miRNAs-carried by circulating exosomes to operate as biomarkers in a variety of diseases has been documented. For example, exosomes containing miR-133a, miR-143/145, miR-150, miR-155, miR-214, miR-223 and miR-320b predict early stage myocardial damage [115] and the outcome of the vascular inflammation and atherosclerosis [114].

Plasma MVs carrying miR-129-5p are a sensitive and specific biomarker for heart failure in univentricular heart disease, independent of ventricular morphology [116]. Moreover, the level of miR-126 or miR-199a expression in circulating MVs could predict the occurrence of cardiovascular events in stable coronary artery disease patients [117]. Also, we found that in atherosclerotic platelet-derived MVs, miR-222, miR-221, miR-210 and miR-34a were significantly increased, whereas the expression of miR-223, miR-214, miR-146a, miR-143, miR-10a and miR-145 was significantly decreased [118].

All of these studies advance the idea that circulating EVs could be helpful as alternative liquid biomarkers for the diagnosis, monitoring and evaluation of treatments in atherosclerosis and associated diseases, like thrombosis. This makes EVs an attractive therapeutic agent and target. However, for the use of EVs as diagnostic tools or therapeutic agents, high-precision isolation and characterization technology is required [119,120].

### 4.4. Extracellular Vesicles Deliver Molecules to Target Cells Acting as Therapeutic Agents

A growing number of studies have focused on the use of cell-derived EVs or engineered EVs to carry drugs to target cells. To devise a drug delivery system, a key constituent is the carrier, that should package and protect the cargo molecules, exhibit low immunogenicity and good stability in the body fluids, and deliver the cargo to the target site. To a great extent, EVs respond to these requirements. They are naturally occurring nanovesicles, and as such, have low immunogenicity and toxicity, are biocompatible, can package proteins, lipids, DNA, RNA and deliver to recipient cells. Moreover, EV delivery of miRNAs, mRNAs and siRNAs could have a variety of applications in gene therapy and drug discovery. It has been reported, in a rat model, that exosomes derived from human synovial mesenchymal stem cells (MSCs) overexpressing miR-140-5p increase cartilage tissue regeneration and prevent knee osteoarthritis. Exosomes derived from dendritic cells overexpressing miR-146a suppress the effects of myasthenia gravis. In addition, exosomes derived from engineered MSCs overexpressing miR-let7c have antifibrotic activity and reduce kidney injury [121].

Furthermore, the combination of exosomes using RNA interference technology is a promising approach for gene therapy. Thus, plasma-derived exosomes have been used as gene delivery platforms to transfer exogenous siRNAs to monocytes and lymphocytes, silencing the target MAPK gene. Likewise, HeLa cell-derived exosomes transfected with siRNA knocked down the target gene RAD51 in the recipient cells [122]. The delivery of a nonviral mini-circle plasmid carrying HIF-1α into the endothelium of mouse ischemic myocardium was shown to induce the release of endothelial cell-derived exosomes enriched in miR-126 and miR-210. This led to the activation of prosurvival kinases, an increase of angiogenic responses and a decrease of metabolic demand, improving tolerance against ischemic stress [123].

These reports indicated that exosomes could be used as therapeutic agents to modulate target genes for therapeutic interventions, and for future combinations of gene- and cell-based therapies. Definitively, supplementary investigations are required to develop efficient strategies involving exosomes.

Concerning MVs, a growing body of data has revealed the role and significance of cargo miRNAs in varied biological and pathophysiological conditions, supporting their therapeutic potential.

It has been reported that the systemic treatment of *ApoE*^−/−^ mice with endothelial cell-derived MVs carrying miR-222 diminished ICAM-1 expression in recipient endothelial cells [124]. Endothelial cell-derived EVs transporting KLF2, which binds to promoters, inducing miR-143/145 cluster upregulation, were shown to control target gene expression in SMCs and reduce atherosclerotic lesion formation in the aorta of *ApoE*^−/−^ mice [125].

Recently, in an atherosclerotic hamster model, we found that intravenous administration of allogenic cell-derived MVs of healthy origins defended against atherosclerotic cardiovascular disease via the transfer of miR-223, miR-21, miR-126 and miR-146a to circulating late endothelial progenitor cells (EPCs), recovering left ventricular and vascular wall function. This showed that EPC-derived MVs reproduced the functions of their parent cells of healthy origins, possibly via miRNA transfer. The beneficial effects of EPC-derived MVs in atherogenesis were found to be greater than those of circulating MVs [126].

In another study, it was reported that EPC-MVs increased the proliferation rate of renal tubular cells and reduced apoptosis and reactive oxygen species of human brain microvascular endothelial cells under hypoxia-reoxygenation [59].

Notably, platelet-derived MVs carrying P-Selectin on their surface upon binding its ligand, PSGL-1, improved the kinetics of fibrinogenesis and normalized the bleeding time of hemophilia mice by targeting the injured site [127].

Recently, special attention was paid to the role of stem-cell derived MVs in cell-free-based therapy for microvascular diseases. Thus, in mice with diabetic retinopathy treated with CD34^+^-cell (EPC)-derived MVs, carrying miR-130a and miR-126 delivered to an ischemic area is considered the main mechanism underlying the preservation of the vascular function. In addition, MSC-derived MVs inhibited vascular remodeling by transferring miR-126 and miR-22 and enhancing the migration of microvascular EC by delivering miR-146a and miR-22 into the cell cytosol [128]. Direct injection into the mouse retina of MVs from ischemic preconditioned-MSCs had a protective effect, substantially reducing ischemia and apoptosis by transferring miR-22 to surrounding cells [129].

Although significant progress has been made in the study of the therapeutic potential of EVs carrying miRNAs, there are still several unanswered questions. For instance, as a function of cell type specificity, the same miRNA could exhibit opposite roles. The prediction of false-positive and false-negative miRNA targets remains a concern. More basic and clinical investigations are required to establish reliable sources of EVs for therapeutic use. The mechanisms for loading functional molecules into exosomes and the best carrier-vesicles are yet to be determined.

*Engineered exosomes as vehicles for biologically active proteins.* Recently, engineered EVs, considered to be naturally occurring nanovesicles, were designed for the transfer of cargo proteins/RNAs/drugs to different tissues and cells. They represent an important step in the development of EV-based therapeutics. For example, engineered exosomes were used as vehicles to deliver biologically active proteins to the brain. Using WW-Cre reporter exosomes, it was shown that nasal delivery resulted in the transport of the cargo-carried EVs across the blood-brain barrier to several brain regions including the olfactory bulb, cortex, striatum, hippocampus and cerebellum [130].

Also, the systemic administration of engineered M2 anti-inflammatory macrophage-derived exosomes (M2 Exo) further electroporated with FDA-approved hexyl 5-aminolevulinate hydrochloride (HAL) displayed inflammation-tropism and anti-inflammatory effects via the surface-exposed chemokine receptors and the anti-inflammatory cytokines released from the anti-inflammatory M2 macrophages [131].

## 5. COVID-19-Associated Thrombosis and Extracellular Vesicles

### 5.1. Thrombosis Is a Common Occurrence in Corona-Virus Disease 19 (COVID-19)

COVID-19 (Corona-Virus Disease 19) is a new infectious disease that grew into a major human health problem with catastrophic global impact. It is caused by the severe acute respiratory syndrome-coronavirus-2 (SARS CoV-2), a positive-sense, single-stranded RNA virus that exhibits membrane proteins, spike proteins, nucleocapsid proteins and envelope proteins. The virus uses the cellular angiotensin-converting enzyme 2 receptor (ACE2) for internalization, aided by transmembrane protease, serine 2 (TMPRSS2 protease) [132].

ACE2, that mediates the internalization of SARS-CoV2, is highly expressed on numerous human cells, including type II alveolar cells, endothelial cells, nasal, oral, esophageal, ileal epithelial cells, myocardial cells and the kidney proximal tubule cells [133].

COVID-19 and the associated acute respiratory distress syndrome (ARDS) causes diffuse lung alveolar and endothelial cell damage with severe inflammation, increased vascular permeability and poor pulmonary oxygenation. The histopathological findings of lungs conducted on deceased COVID-19 patients revealed the presence of numerous fibrin thrombi in small vessels, i.e., capillaries and intra-alveolar fibrin depositions [134,135].

In addition to systemic inflammation and respiratory disease, thrombosis is a common occurrence in COVID-19 patients, causing significant morbidity and mortality [136].

One prospective cohort study indicated that COVID-19 positive cancer patients with cardiovascular disease have increased mortality compared to COVID-positive cancer patients without comorbidities [137].

ACE2 receptor is a carboxypeptidase that converts angiotensin II (Ang II) to angiotensin 1–7. The binding of the viral S-protein to ACE2 is followed by internalization and downregulation of the receptor and a subsequent increase in Ang II as a result of the disturbed balance between the prothrombotic Ang II and and the anti-thrombotic angiotensin 1–7 signalling [138]. Besides the dysregulated renin angiotensin aldosterone system, it is assumed that two other mechanisms are implicated in COVID-19-associated thrombosis, namely, excess cell secretion of vWF and the activation of the innate immune response. In the case of the former, injury to endothelial cells may lead to the accumulation of vWF in the subendothelial space, that acts as a molecular glue between platelets and the subjacent collagen, leading to platelet activation, aggregation and thrombosis [139,140]. Also, an uncontrolled innate immune response elicited by over-activated neutrophils could initiate coagulopathic pathways, entailing excessive complement activation, cytokine storm and NETosis, each of which may cause thrombosis [141].

**Figure 3:**
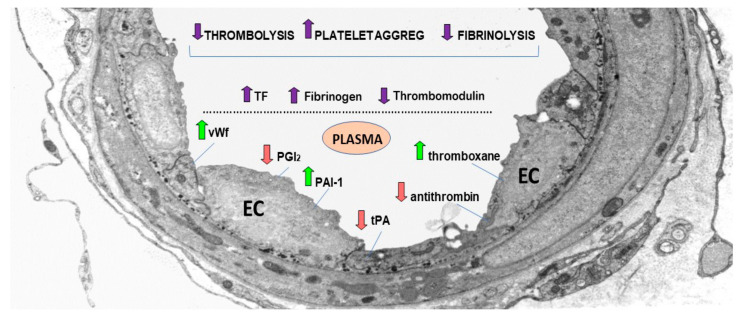
Thrombosis is a consequence of reduced fibrinolysis and thrombolysis processes, and the subsequent dramatic increase in platelet aggregation due to an imbalance of endothelial and platelet circulating mediators. The figure shows an electron-micrograph of an arteriole exhibiting activated endothelial cells (EC), and the upregulated and downregulated mediators (arrows) that lead to thrombosis. An increase in plasma von Willebrand factor (vWF) that enhances platelet adhesivity, plasmin activator inhibitor (PAI-1) that inhibits tPA and uPA, and thromboxane A2 that activates the platelets, leads to platelet aggregation and the formation of thrombus. Concomitantly, thrombus formation is due to a decrease in plasma prostacyclin (PGI2) that controls platelet activation, tissue plasmin activator (tPA) that, together with urokinase plasminogen activator (uPA), breaks down fibrin into fibrin degradation products, and antithrombin (an active anticoagulant). Ultimately, the increase in tissue factor (TF) and fibrinogen, and the decrease of thrombomodulin, create a prothrombotic milieu by initiating the coagulation cascade, platelet aggregation and the formation of thrombus.

It has been suggested that the highly elevated levels of D-dimer and fibrin degradation product (FDP) could be associated with COVID-19 progression [142]. Other studies considered that COVID-19 patients are at increased risk of thrombosis due to the vasoconstriction induced by high levels of angiotensin II, inflammation and activation of the complement system. Notably, SARS-CoV-2 infection induces the release of TF-positive EVs in the circulation that, in turn, activates epithelial cells, macrophages, neutrophils, platelets and endothelial cells and contributes to the common occurrence of thrombosis in COVID-19 patients [143]. The vascular endothelium exhibits a markedly reduced thrombolysis and fibrinolysis and a significant increase in platelet aggregation due to an imbalance between the major mediators implicated in these processes (Figure 3).

### 5.2. Extracellular Vesicles as Biomarkers in COVID-19-Associated Thrombosis

The role of EVs in infectious diseases has been particularly controversial over recent years. It has been shown that EVs can influence the activities of the recipient cell by transporting viral proteins, RNA, DNA and receptors from infected cells to healthy cells, thus increasing the spread of viral infection. For example, it has been reported that in human papillomavirus, EVs deliver miRNAs to unaffected cells, thus contributing to the progression of cervical inflammation [144]. Other researchers have hypothesized that EVs may negatively regulate viral infection through cytokine secretion that could induce immune system responses against viral pathogens [145].

The identification of specific biomarkers is vital for the prevention, evolution and clinical decisions concerning COVID-19. As mentioned above, EVs contain numerous and diverse biomolecules, and their cargoes can be modified by microenvironmental stimuli including viral infection. EVs exhibit surface molecules, such as CD9 and ACE2 [146]. Thus, Fujita et al., hypothesized that serum EVs may serve as potential predictors of COVID-19 severity. They analyzed EV proteins, including coagulation-related markers and antiviral response-related EV proteins with the potential to serve as early predictive biomarkers for COVID-19 severity. Analyses of proteome profile by liquid chromatography mass spectrometry of EVs collected from 31 SARS-COV-2 infected patients and 10 healthy donors allowed the authors to identify significant differences in the EV-cargo. They found that fibrinogen gamma chain (FGG), CD147, calpain 2 (CAPN2), extracellular matrix protein 1 (ECM1), coat complex subunit beta 2 (COPB2), KRAS proto-oncogene (KRAS), protein kinase C beta (PRKCB) and ras homolog family member C (RhoC) were significantly more abundant in SARS-COV-2 infected patients than in healthy donors. This group of markers can distinguish between severe and mild cases of COVID-19; among them, COPB2 has the best predictive value [147].

Interestingly, the levels of circulating EVs and the associated TF expression (EV-TF) were shown to be significantly higher in patients with COVID-19 compared to controls. TF expression is tightly implicated in the activation of coagulation and thrombosis. There are several mechanisms underlying the increase in TF expression during viral infection. In a clinical study on 100 patients with COVID-19, it was found that EV-TF activity correlated positively with D-dimer, prothrombin time (PT), international normalized ratio (INR) calculated based on the PT test result (PT/INR), prothrombin, fibrinogen and antithrombin. Therefore, the circulating EV levels and EV-TF activity could be used as prognostic biomarkers in patients with COVID-19 [148].

Recently, in a cohort of 111 hospitalized patients with COVID-19, the EV-TF activity was shown to correlate positively with the inflammatory state, disease severity and thrombotic events. This study recommends systematic preventive anticoagulation in patients hospitalized with COVID-19, and potential intensification of anticoagulation in patients with severe disease [149]. A significantly higher level of circulating platelet-derived MVs in COVID-19 patients in comparison with healthy subjects was reported [150]. Interestingly, although the concentration of platelet-derived MVs was increased, the number of circulating platelets did not change between the two cohorts. This indicates that circulating platelet-derived MVs are not only significantly correlated with Sars-Cov-2 infection, but that they may be used as diagnostic biomarkers of viral infection. Reportedly, they contain elevated levels of coagulation factors and immune mediators that can induce platelet aggregation, mediate the coagulation pathway and activate enzymes such as cyclooxygenase-1 and 12-lipoxygenase [151]. Although these studies provided candidate biomarkers, future investigations should focus on elucidating their pathogenic role in venous thrombosis in COVID-19 patients.

Regarding viral transmission, it has been shown that EVs from patients infected with COVID-19 transfer to target cells prothrombotic and endothelial injury factors such as TF, t-PA, vWF, proteins associated with cardiovascular pathology (MB, PRSS8, REN, HGF), cytokines (TNF-α, IL-6), chemokines (MCP-1, CXCL16), proteases and peptidases including cathepsin L1, an enzyme involved in tissue remodelling [152]. Moreover, using an in vitro approach, the authors showed that EVs from patients infected with COVID-19 contributed to increased caspase 3/7 activity, leading to apoptosis of pulmonary endothelium. Their experiments on human pulmonary microvascular EC revealed that EVs isolated from the plasma of severely diseased patients play an essential role in the pathogenesis of COVID-19.

Recently, the presence of SARS-CoV-2 RNA in the circulating exosomal cargo was identified, suggesting that the virus could use the endocytosis route to spread. Thus, circulating exosomes may play an essential role in inflammation, coagulation and immunomodulation during SARS-CoV-2 infection [153].

The essential role of NLRP3 inflammasome is known in the activation of the inflammatory cascade involving cytokines (i.e., IL-1β, IL-6, TNFα) and other mediators as part of the host inflammatory responses to SARS-CoV infection. We can speculate that infected exosomes carying SARS-CoV-2 RNA could activate the NLRP3 inflammasome. The possible activation of the NLRP3 inflamasome by SARS-COV and its possible therapeutic target in COVID-19 disease was discussed by Shah [154]. There is a relationship between inflammasome activation and hypercoagulopathy in COVID-19 patients [155].

Hyperglycemia also synergize with SARS-CoV-2, contributing to a deleterious hyperinflammation in diabetic patients affected by COVID-19 [156]. This reaction could also apply to athero-diabetic patients infected with SARS-CoV-2.

More monitoring of exosomal content during SARS-COV-2 infection is needed to clarify the role played by exosomes in viral spread and immunological protection.

The table below (Table 1) summarizes some recent results on the use of EVs as biomarkers.

### 5.3. Extracellular Vesicles as Therapeutic Agents in COVID-19-Related Thrombosis

Due to their stability in the circulation, low immunogenicity, biocompatibility and biodegradation, EVs are considered suitable for designing new therapeutic strategies or delivery systems for a vaccine against the SARS CoV-2 infection [157].

As for the therapeutic ability of EVs, a systematic review of the literature (39 articles published 2014–2020) reported the beneficial effects in pneumonia, pulmonary fibrosis and ARDS pathogenesis [158]. This review shows that EVs derived from stem cells may be beneficial in treating lung injury, reducing inflammation, promoting repair of alveolar and bronchiolar epithelial cells and preventing pulmonary fibrosis. The therapeutic role of EVs was attributed to the miR-126, miR-30b-3p, miR-145, miR-27a-3p, syndecan-1, hepatocyte growth factor and angiopoietin-1. All these biomolecules promote modulation of innate immune responses, epithelial-mesenchymal transition for lung regeneration, attenuation of lung permeability and inflammation [159,160,161,162]. Based on these data, we can safely assume that EVs will be the next target for the initiation of innovative therapies against COVID-19.

It is not known whether the administration of EVs would function in the context of disseminated intravascular thrombosis. A few studies have shown that intravenously (i.v.) administered EVs are capable of reaching the inflamed lungs. Since patients with severe COVID-19 infection often suffer from multiple organ failure, intravenously administrated EVs could be beneficial to treat several affected organs, in addition to the lungs [163].

In this context, a study performed on 24 patients with COVID-19 found that exosomes derived from allogeneic bone marrow mesenchymal stem cells (Exo-MSC) are promising therapeutic candidates for severe COVID-19, by reducing the circulating CRP, ferritin and D-dimer [164]. The latter, a product of fibrin degradation, is positively correlated with thrombotic complications and may be used as a prognostic biomarker in patients with COVID-19 [142,165].

Importantly, a recent study indicated that one i.v. treatment with Exo-MSC and oxygenation improved the clinical status of COVID-19 patients, showing an average increase in PaO_2_/FiO_2_ ratio of 192% [164]. The severity of this disease led to other therapeutic strategies, such as the aerosol inhalation of the Exo-MSC.

At present, on ClinicalTrials.gov, there are three registered clinical trials using inhalation of Exo-MSC for the treatment of COVID-19 (Identifier: NCT04276987, NCT04491240, NCT04602442). As study is ongoing, the results have not yet been published.

In a recent study that was successfully completed (phase 1 trial), it was found that exosomes isolated and purified from T-REx™-293 cells engineered to express CD24 at high levels (EXO-CD24) inhaled once a day for a few minutes for five days selectively restrained certain cytokine and led to recovery in 35 patients with COVID-19 whose conditions were moderate or worse (ClinicalTrials.gov Identifier: NCT04747574). Overall, studies regarding the therapeutic role of EVs in COVID-19 require an in-depth understanding of EV safety and efficacy.

Finding reliable clinical biomarkers from biological fluids will be the next crucial challenge for the development of new therapeutic strategies against COVID-19. The table below (Table 2) summarizes the latest results concerning the use of EVs as therapeutic agents in COVID-19.

## 6. Conclusions and Future Directions

Accumulated evidence, including our own, has led to the perception that EVs function as effective vectors of biological material, protagonists of intercellular communication, signaling mediators, and promising prognostic biomarkers and therapeutic agents in various diseases, including atherosclerosis and COVID-19-related thrombosis.

Cells use EVs as biological tools to communicate and transfer matter from donor cells to recipient cells. Upon transfer, cargo molecules (DNA, mRNA, miRNA and others) affect and influence the physiology or/and pathophysiology of the receiver cell.

In atherosclerosis, and even more so in COVID-19 disease, EVs could have either a harmful or a protective effect. Thus, depending on the cargo and the microenvironment, EVs may promote the propagation of atherosclerotic plaque or COVID-19-related thrombosis. Alternatively, EVs could be a reliable biomarker and signal disease progression. Consequently, EVs could be employed as a therapeutic agent. In this respect, engineered EVs might represent a novel therapeutic tool in cardiovascular medicine and regenerative therapy. Although important advances have been made in EV science, there is yet much to be discovered. More data are needed on their capacity to select biological content, the mechanisms of interaction with target cells and the implications in physiological and pathophysiological processes. We have to clarify the capacity of cells to produce EVs of different sizes and compositions. An explanation is needed for the selection by which EVs, during their biogenesis, selectively encapsulate either DNA, RNAs, proteins or lipids, characteristics that are part of the EV molecular signature. Since the cargo is usually in small quantities, there is a question about whether this is sufficient to explain its biological effects. More experiments may reveal how EVs recognize target (recipient) cells and how the cargo is processed upon cell endocytosis. Also, we hope to find whether a connection exists between the size, the content and the cargo of EVs and the stage of the disease. Moreover, there is an urgent need to validate results obtained in cultured cells in vivo.

There are still a great number of questions to be answered. But this is the beauty of science; the more one discovers, the more questions arise.

## Figures and Tables

**Table 1:** Changes in the molecular composition of extracellular vesicles (EVs) isolated from COVID-19 patients, on the basis of which they could serve as biomarkers for the severity of the disease and associated thrombosis. PMVs: platelet-derived MVs; EV-TF: EVs and associated TF; exRNA: extracellular RNA; 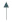 increase.

Extracellular Vesicles (EVs) as Prognostic and Biomarkers in COVID-19 and Related Thrombosis			
EV Isolated from Serum or Plasma of COVID-19 Patients	Extracellular Vesicles– Molecular Changes	EVs as Prognostic and Biomarkers in COVID-19 Patients	References
**EVs from serum** (31 COVID-19 patients (22 with mild and 9 with severe symptoms)/10 healthy subjects (control))	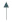 COPB2/KRAS/PRKCB/RHOC expression in patients with mild COVID-19 symptoms 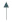 CD147/CAPN2/ECM1/FGG expression in patients with severe COVID-19 symptoms 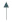 exRNA:SNORD33/AL732437.2/RNU2-29P/CDKN2B-AS1/ AL365184.1/miR-122-5p expression in patients with severe COVID-19 symptoms	- essential role in distinguishing severe and mild forms of COVID-19 - role in developing an effective therapeutic plan for early disease prediction	[147]
**Plasma EVs** (100 COVID-19 patients (with mild and severe symptoms)/28 healthy subjects (control))	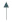 TF-positive EV levels in COVID-19 patients 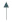 EV-TF activity in COVID-19 patients 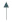 EV-TF activity—positively correlated with plasma markers: D-dimer, PT, INR, prothrombin, fibrinogen, antithrombin, vWF (markers directly associated with thrombosis)	- contribute to thrombosis in patients with COVID-19 - EV-TF activity—prognostic biomarker in patients with COVID-19	[148,149]
**Blood PMVs** (69 COVID-19 patients, 62 patients after COVID-19, 10 healthy subjects)	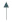 PMV levels in COVID-19 patients - platelet levels unchanged in COVID-19 patients compared to healthy subjects	- positively correlated with Sars-CoV-2 infection - can be used as a biomarker to diagnose Sars-Cov-2 infection	[150]
**EVs from plasma** (53 COVID-19 patients (with mild and severe symptoms)/healthy subjects (control))	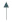 PMV levels in patients with mild COVID-19 symptoms 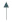 exosome levels in patients with severe COVID-19 symptoms 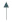 TF/t-PA/vWF/CD163/EN-RAGE expression (prothrombotic/endothelial injury factors) in COVID-19 patients with severe pathology 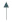 HGF/MB/REN/prostasin/PRSS8 (factors associated with cardiovascular pathology) in COVID-19 patients with severe pathology 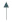 TNF-α/IL-6/MCP-1 levels in patients with mild and severe COVID-19 symptoms 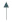 caspase 3/7 activity in patients with mild and severe COVID-19 symptoms	- important role in assessing the severity of COVID-19 patients - can contribute to identifying specific markers of endothelial injury and inflammation in COVID-19 patients	[152]

**Table 2:** Preliminary results in the assessment of the therapeutic role of exosomes in patients with COVID-19. EVs: extracellular vesicles; MVs: microvesicles; PMVs: platelet-derived MVs; PaO_2_/FiO_2_ ratio: arterial pressure of oxygen/inspired fraction of oxygen ratio; 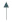 increase; 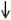 decrease.

Employing Extracellular Vesicles (EVs) as Therapeutic Agents in COVID-19			
Administration of MSC-Derived EVs to Patients	Improved Effects	EV Effects in COVID-19 Patients	References
**MSC-derived exosomes** administered i.v. to 24 COVID-19 patients with moderate to severe pathology	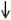 CRP/ferritin/D-dimer levels 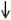 neutrophil number 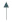 CD3^+^/CD4^+^/CD8^+^ lymphocyte number 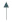 PaO_2_/FiO_2_ ratio	- restored oxygenation - downregulate cytokine storm; - promising therapeutic candidates for severe COVID-19	[164]
**MSC-derived exosomes** administered by aerosol inhalation to COVID-19 patients: 5 days to 24 patients; twice a day, 10 days to 30 patients	No official results yet		ClinicalTrials.gov: NCT04276987/ NCT04491240/ NCT04602442
**Engineered exosomes** overexpressing CD24, aerosolized by inhalation, once a day, 5 days to 35 COVID-19 patients	suppress cytokine storm	- recovery of patients with moderate or worse condition	ClinicalTrials.gov: NCT04747574

## Data Availability

Not applicable.

## Abbreviations

ABCA1	ATP binding cassette A1
ABCG-1	ATP-binding cassette sub-family G member 1
ACE2	angiotensin-converting enzyme 2 receptor
Akt	protein kinase B
ALIX	apoptosis-linked gene 2-interacting protein X
ApoBDs	apoptotic bodies
ARDS	acute respiratory distress syndrome
ARF6	ADP-ribosylation factor 6
CAPN2	calpain 2
CCL5	chemokine (C-C motif) ligand 5
CHIP	clonal hematopoiesis of indeterminate potential
COPB2	coat complex subunit beta 2
COVID-19	coronavirus disease-2019
CRP	C-reactive protein
DLL4	Notch ligand Delta-like 4
E	envelope proteins
ECM1	extracellular matrix protein 1
ECs	endothelial cells
EGFRs	epidermal growth factor receptors
EMVs	endothelial-derived microvesicles
eNOS	endothelial nitric oxide synthase
EpCAM	epithelial cell adhesion molecule
EPCs	endothelial progenitor cells
ERK	extracellular regulated protein kinase
ESCRT	endosomal sorting complexes required for transport
ESEs	early sorting endosomes
EVs	extracellular vesicles
Exo-MSC	bone marrow mesenchymal stem cells
FDP	fibrin degradation product
FGG	fibrinogen gamma chain
Hsp	heat-shock proteins
ICAM-1	intercellular adhesion molecule-1
IFNγ	interferon gamma
IL-1	interleukin-1
ILVs	intraluminal vesicles
INR	international normalized ratio
L1CAM	L1 cell adhesion molecule
LC-MS	liquid chromatograph mass spectrometers
LFA-1	lymphocyte function-associated antigen 1
LMVs	leukocyte-derived microvesicles
LSEs	late sorting endosomes
M	membrane proteins
M2 Exo	M2 macrophage-derived exosomes
Mac-1	macrophage integrin-1
MCP-1	monocyte chemoattractant protein-1
M-CSF	monocyte colony stimulating factor
miRNA	microRNA
MMP2	matrix metallopeptidase2
MPs	microparticles
MSCs	mesenchymal stem cells
MVBs	multivesicular bodies
MVs	microvesicles
N	nucleocapsid proteins
ncRNA	non-coding RNAs
NLRP3	nod-like receptor protein 3
oxLDL	oxidized low-density lipoproteins
PDGF	platelet-derived growth factor
PDGF	platelet-derived growth factor
PMVs	platelet-derived microvesicles
PPAR-γ	proliferator-activated receptor-γ
PRKCB	protein kinase C beta
PS	phosphatidylserine
PSGL-1	P-Selectin glycoprotein ligand 1
PT	prothrombin time
RhoC	ras homolog family member C,
S	spike proteins
SARS-CoV-2	severe acute respiratory syndrome coronavirus-2
SDF-1α	stromal cell-derived factor 1 α
SMCs	smooth muscle cells
TF	tissue factor
TGF-β	transforming growth factor β
TMPRSS2	transmembrane protease, serine 2
TNFα	tumour necrosis factor α
Th-1	T helper 1 cells
T-reg	regulatory T cells
TSG101	tumor susceptibility gene 101
VCAM-1	vascular-cell adhesion molecule-1
VEGF	vascular endothelial growth factor
vWF	von Willebrand factor
